# Prognostic value of consolidation-to-tumor ratio on computed tomography in NSCLC: a meta-analysis

**DOI:** 10.1186/s12957-023-03081-y

**Published:** 2023-06-22

**Authors:** Yongming Wu, Wenpeng Song, Denian Wang, Junke Chang, Yan Wang, Jie Tian, Sicheng Zhou, Yingxian Dong, Jing Zhou, Jue Li, Ziyi Zhao, Guowei Che

**Affiliations:** 1 Department of Thoracic Surgery, West China Hospital, Sichuan University, Chengdu, Sichuan China; 2 Lung Cancer Center, West China Hospital, Sichuan University, Chengdu, Sichuan China; 3 Precision Medicine Center, Frontiers Science Center for Disease-Related Molecular Network, West China Hospital, Sichuan University, Chengdu, 610041 Sichuan China; 4 Department of Respiratory and Critical Care Medicine, Frontiers Science Center for Disease-Related Molecular Network, West China Hospital, Sichuan University, Chengdu, 610041 Sichuan Province China

**Keywords:** Non-small cell lung cancer, Consolidation to tumor ratio, Prognostic, Meta-analysis

## Abstract

**Background:**

Although several studies have confirmed the prognostic value of the consolidation to tumor ratio (CTR) in non-small cell lung cancer (NSCLC), there still remains controversial about it.

**Methods:**

We systematically searched the PubMed, Embase, and Web of Science databases from inception to April, 2022 for eligible studies that reported the correlation between CTR and prognosis in NSCLC. Hazard ratios (HRs) with 95% confidence intervals (95% CIs) were extracted and pooled to assess the overall effects. Heterogeneity was estimated by *I*^2^ statistics. Subgroup analysis based on the cut-off value of CTR, country, source of HR and histology type was conducted to detect the sources of heterogeneity. Statistical analyses were performed using STATA version 12.0.

**Results:**

A total of 29 studies published between 2001 and 2022 with 10,347 patients were enrolled. The pooled results demonstrated that elevated CTR was associated with poorer overall survival (HR = 1.88, 95% CI 1.42–2.50, *P* < 0.01) and disease-free survival (DFS)/recurrence-free survival (RFS)/progression-free survival (PFS) (HR = 1.42, 95% CI 1.27–1.59, *P* < 0.01) in NSCLC. According to subgroup analysis by the cut-off value of CTR and histology type, both lung adenocarcinoma and NSCLC patients who had a higher CTR showed worse survival. Subgroup analysis stratified by country revealed that CTR was a prognostic factor for OS and DFS/RFS/PFS in Chinese, Japanese, and Turkish patients.

**Conclusions:**

In NSCLC patients with high CTR, the prognosis was worse than that with low CTR, indicating that CTR may be a prognostic factor.

**Supplementary Information:**

The online version contains supplementary material available at 10.1186/s12957-023-03081-y.

## Introduction

An estimated 1.8 million cancer-related deaths were recorded in 2020 due to lung cancer, according to the latest global statistics on cancer [1]. Most of the pathologic subtypes of lung cancer are NSCLC, in which adenocarcinoma accounts for a large proportion [2]. Although many cases can be detected early and the treatment has been significantly improved, patients with lung cancer continue to face unsatisfactory prognoses, due to metastasis or recurrence [3].

Recently, with the extensive use of chest computed tomography (CT), many lung cancers have been detected to contain ground-glass opacity (GGO), which was defined as an area of a slight, homogenous increase in density without obscuring the underlying vascular markings on CT in previous studies [4]. Several studies have demonstrated that GGO components on CT indicated improved survival in NSCLC, especially in lung adenocarcinoma [5, 6]. The relationship between preoperative radiological findings, such as the maximal standardized uptake value (SUVmax), and tumor disappearance ratio, and the prognosis of NSCLC has attracted close attention, due to improvements in imaging technology [7–9].

The consolidation to tumor ratio, which was calculated as the ratio of the maximum consolidation size to the maximum tumor size measured using the lung window setting on CT in several studies, has been used to select patients for sublobar resection or to predict the prognosis of NSCLC patients [10–12]. There are, however, controversies over the prognostic value of CTR in NSCLC. Kim and his colleagues found that CTR was not an independent prognostic indicator for lung adenocarcinoma patients treated with surgery [13]. Xi et al. confirmed the prognostic value of CTR in lung adenocarcinomas [12]. To assess its prognostic value in NSCLC, we performed this meta-analysis.

## Materials and methods

### Registration

Our study has been registered in the International Prospective Registry of Systematic Reviews (PROSPERO) (registration number: CRD42022360462). Details of the protocol can be accessed at https://www.crd.york.ac.uk/prospero/display_record.php?ID=CRD42022360462.

### Literature retrieval

Relevant studies were collected through systematic searches of the PubMed, Embase and Web of Science databases up to April, 2022. The following MeSH terms were used: “cancer”, “tumor”, “neoplasm”, “carcinoma”, “lung”, “pulmonary”, and “consolidation to tumor ratio”. Additionally, references of all included studies and relevant review articles were searched for available articles.

### Inclusion and exclusion criteria

Inclusion criteria: (1) patients were clearly grouped according to the CTR value. (2) Retrospective or prospective studies evaluating the prognostic relationship between CTR and NSCLC. (3) The hazard ratios of OS and/or DFS/RFS/PFS with 95% CIs were reported or sufficient data were obtained to calculate them. (4) NSCLC was confirmed by postoperative pathology. (5) Full-texts were available.

Exclusion criteria: (1) overlapping studies; (2) reviews, case reports, editorials, conference abstracts, or animal trials; (3) the HR or 95% CIs were not available.

### Data extraction and quality assessment

Two researchers (Yongming Wu and Wenpeng Song) independently screened the literature. Any disagreement that arose during the study was resolved through team discussion. The following information was extracted: first author, year of publication, country, study time, sample size, median follow-up time, histology type, TNM stage, clinical outcome, cut-off value of CTR, HR, and 95% CIs. The HR information was recorded directly or gathered from Kaplan‒Meier curves using Engauge Digitizer Version 4.1.

The quality of all eligible studies was evaluated by two researchers (Yongming Wu and Wenpeng Song) using the Newcastle‒Ottawa quality assessment scale (NOS). A study was considered high quality if it had an NOS score of 6 or greater.

### Statistical analysis

All statistical analyses were conducted using Stata 12.0 software. The pooled HRs of OS or DFS/PFS/RFS and 95% CIs were used to evaluate the relationship between CTR and prognosis in NSCLC. I^2^ statistics were used to evaluate the heterogeneity. When *I*^2^ > 50% and/or *P* < 0.1, there was obvious heterogeneity, and the random effect model was used, otherwise, the fixed effect model was used [14]. Subgroup analysis based on the cut-off value of CTR, country, source of HR and histology type was performed to explore the source of heterogeneity or further demonstrate the results of the meta-analysis. Sensitivity analysis was used to assess the stability of the results in the enrolled studies. Begg's funnel plot and Egger's test were used to detect publication bias [15, 16]. The trim-and-fill method was used if obvious publication bias was detected. *P* values less than 0.05 were considered statistically significant.

## Results

### Literature search

**Fig. 1:**
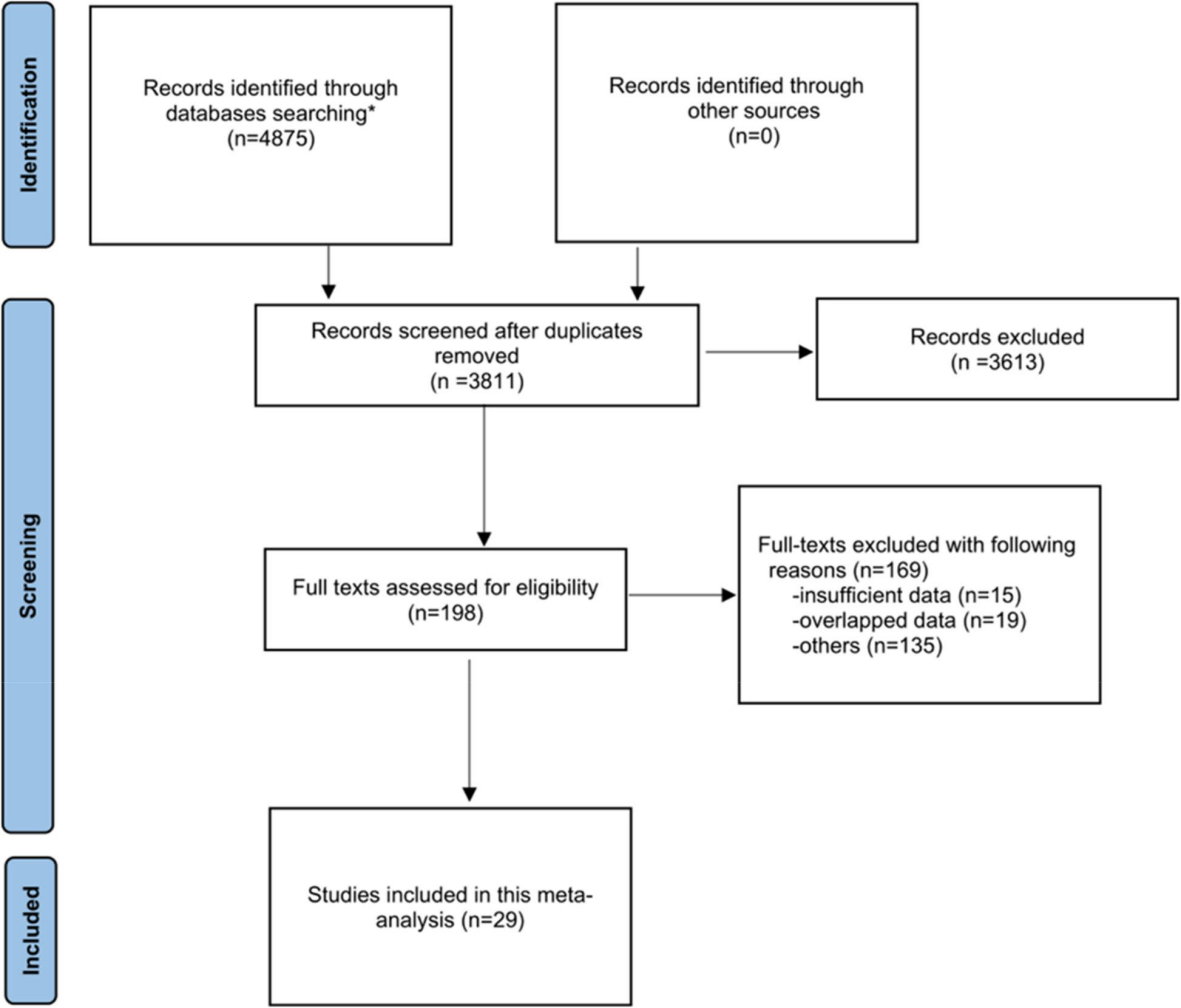
Flowchart of the study search and selection. *PubMed (*n* = 1428), Embase (*n* = 648), Web of Science (*n* = 2799)

According to the search strategies, 4875 articles were retrieved. After duplicates were removed, we carefully read the titles and abstracts of the 3811 studies, and 3613 studies were excluded. Subsequently, 198 potential articles were further evaluated by reading the full text, of which 169 were excluded due to inclusion and exclusion criteria. Finally, 29 qualified studies including 10,347 patients were eligible for pooled analysis [6, 12, 13, 17–42]. The selection process is shown in Fig. 1.

### Characteristics of the included studies

In total, 29 studies published between 2001 and 2022 with 10,347 patients were included. All included studies were retrospective in our study. Most of the studies included were conducted in China and Japan; only two studies originated from Korea (*n* = 2) [13, 21] and one from Turkey (*n* = 1) [30]. All included studies had an NOS score of at least 7 (with a mean value of 7.45), which meant they were all high-quality studies (Supplementary file Table S2). The characteristics of all qualified literature sources are recorded in Table 1.

**Table 1:** Basic characteristics of included studies

**Authors**	**Year**	**Study period**	**Country**	**Sample size**	**MFP** **(months)**	**Study type**	**Histology type**	**Cut**-**off**	**Endpoint**	**Source of HR**	**N**os
**Aoki et al**. [17]	2001	1990–1999	Japan	127	37.1	Retro	ADA	0.5	OS	E	7
**Higashi et al.** [18]	2009	1997–2005	Japan	87	18	Retro	ADA	0.5	DFS	E	7
**Koike et al.** [19]	2012	1992–2009	Japan	223	70	Retro	NSCLC	0.75	OS	R	8
**Kishimoto et al.** [20]	2014	2006–2010	Japan	169	42	Retro	NSCLC	C	DFS	R	8
**Nakamura et al.** [22]	2015	2005–2011	Japan	113	46	Retro	ADA	0.5	OS/DFS	R	8
**Shimada et al.** [23]	2015	2004–2010	Japan	67	58.9	Retro	NSSLC	0.5	OS/RFS	R	8
**Cho et al.** [21]	2015	2001–2010	Korea	97	44.7	Retro	ADA	0.25	OS/RFS	E	8
**Tsurugai et al.** [24]	2016	2005–2014	Japan	155	34.7	Retro	NSCLC	0.5	OS/DFS	R	8
**Suzuki et al.** [25]	2017	2003–2007	Japan	392	84	Retro	ADA	0.5	OS/RFS	E	7
**Tsunezuka et al.** [26]	2017	2008–2012	Japan	62	55.1	Retro	NSCLC	0.5	OS/RFS	R	7
**Huang et al.** [27]	2018	2004–2013	China	789	87	Retro	ADA	0.75	OS/DFS	E	7
**Ye et al.** [28]	2018	2008–2014	China	736	38	Retro	ADA	C	RFS	R	7
**Kamigaichi et al.** [29]	2019	2007–2016	Japan	166	49.3	Retro	NSCLC	0.85	OS/RFS	E	7
**Kim et al.** [13]	2019	2009–2015	Korea	691	39	Retro	ADA	0.5	DFS	R	7
**Ye et al**. [6]	2019	2008–2014	China	329	42.2	Retro	ADA	0.5	OS	R	8
**Kuroda et al.** [31]	2020	2006–2010	Japan	260	83.8	Retro	NSCLC	0.5	OS/DFS	R	7
**Kabalak et al.** [30]	2020	2013–2016	Turkey	156	40	Retro	ADA	0.5	OS/PFS	E	8
**RYOJI IWAMOTO et al.** [33]	2021	2000–2009	Japan	73	77	Retro	ADA	0.8	OS	R	7
**Takamori et al**. [37]	2021	2006–2014	Japan	85	87.6	Retro	NSCLC	0.5	OS	E	7
**Sun et al.** [36]	2021	2014.01–2014.12	China	257	76	Retro	NSCLC	C	OS/RFS	R	7
**Zhong et al.** [39]	2021	2011–2012	China	620	72.4	Retro	ADA	C	OS/RFS	R	7
**Ji et al.** [34]	2021	2014–2015	China	190	51	Retro	ADA	0.5	PFS	R	7
**Lin et al.** [35]	2021	2013–2015	China	372	55	Retro	ADA	0.5	RFS	R	7
**Xi et al.** [12]	2021	2011–2016	China	862	47	Retro	ADA	C	RFS	R	7
**Chiang et al.** [32]	2021	2011–2017	China	1002	43.2	Retro	ADA	0.5	DFS	R	8
**Tsai et al.** [38]	2021	2003–2015	China	149	74	Retro	ADA	0.5	OS/DFS	R	8
**Hattori et al.** [40]	2022	2008–2017	Japan	603	54	Retro	ADA	C	OS	R	8
**Nakao et al.** [41]	2022	2010–2017	Japan	1014	61	Retro	ADA	C	OS	R	8
**Zhai et al.** [42]	2022	2008–2018	China	501	64.8	Retro	ADA	0.75	OS/DFS	R	8

*MFP* median follow-up time, *HR* hazard ratio, *NOS* Newcastle–Ottawa scale, *NSCLC* non-small cell lung cancer, *Retro* retrospective, *ADA* adenocarcinoma, *C* continuous, *OS* overall survival, *DFS* disease-free survival; RFS, recurrence-free survival, *PFS* progression-free survival, *E* estimated, *R* reported, *NA* not available

### Association between CTR and prognosis in NSCLC patients

**Fig. 2:**
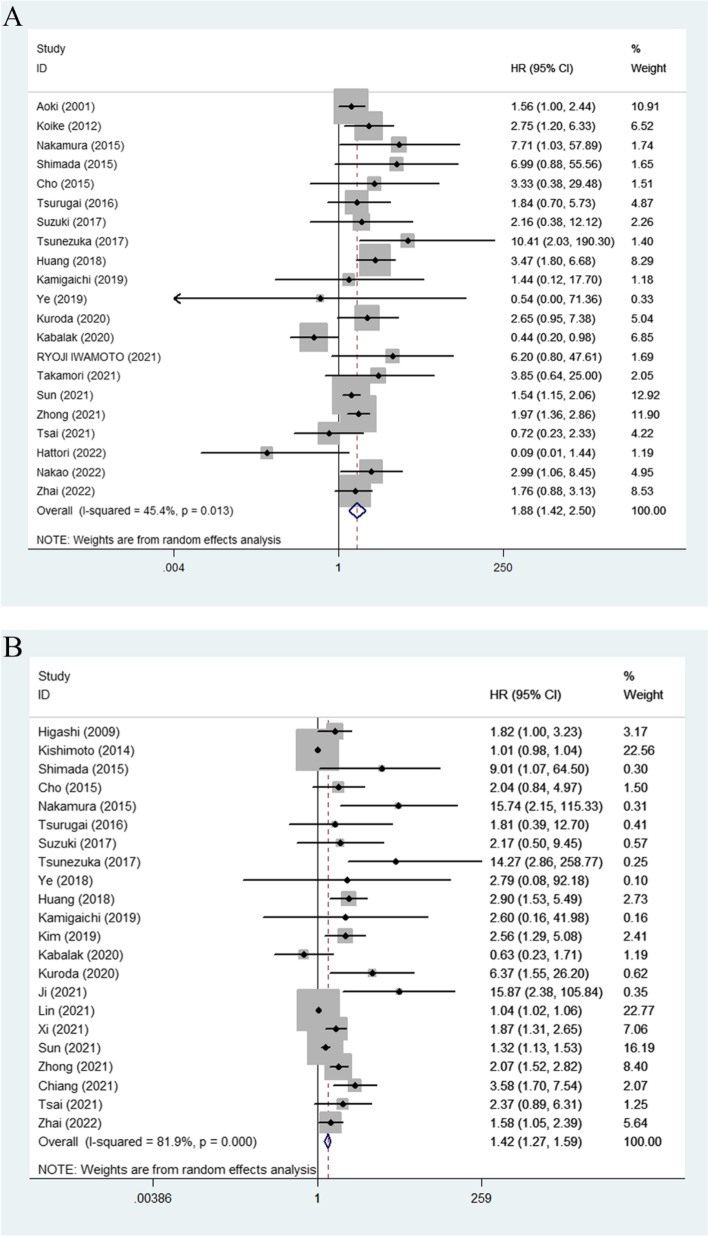
**A** Forest plot for the relationship between CTR and overall survival. **B** Forest plot for the relationship between CTR and DFS/RFS/PFS. CTR, consolidation to tumor ratio; DFS, disease-free survival; RFS, recurrence-free survival; PFS, progression-free survival

The relationship between CTR and OS was reported in 21 studies with 6238 participants [6, 17, 19, 21–27, 29–31, 33, 36–42], and the pooled HR demonstrated that a higher CTR was associated with a worse prognosis (HR = 1.88, 95% CI 1.42–2.50, *P* < 0.01) (Fig. 2A).

There were 22 articles with 7893 participants reporting the impact of CTR on DFS/RFS/PFS [12, 13, 18, 20–32, 34–36, 38, 39, 42], and the results showed that a higher CTR was significantly correlated with poorer prognosis (HR = 1.42, 95% CI 1.27–1.59, *P* < 0.01) (Fig. 2B).

### Subgroup analysis

Subgroup analysis by the cut-off value of CTR indicated that CTR was not a prognostic factor for OS when the cut-off values of CTR were 0.25, 0.8 and 0.85. However, when the cut-off value of CTR was 0.5 or 0.75 or when CTR was a continuous variable, CTR was a prognostic factor for OS. For DFS/RFS/PFS, when the cut-off value of CTR was 0.50 or 0.75 or CTR was a continuous variable, CTR could predict the prognosis of NSCLC patients. Subgroup analysis by country showed that among patients from China, Japan and Turkey, a higher CTR was associated with worse OS and DFS/RFS/PFS. Subgroup analysis stratified by histology type demonstrated that CTR was a predictor for OS and DFS/RFS/PFS in both lung adenocarcinoma and NSCLC patients (Tables 2 and 3). Nine studies had investigated the correlation between CTR and OS in stage I NSCLC patients, and the pooled HR for OS was 1.63 (95% CI 1.05–2.54) (Supplementary file Figure S2). Ten studies were conducted to explore the correlation between CTR and DFS/RFS/PFS in stage I NSCLC patients, the pooled HR for DFS/RFS/PFS was 1.89 (95% CI 1.26–2.85) (Supplementary file Figure S3).

**Table 2:** Subgroup analysis for overall survival

	Number of studies	HR	95% CI	*P* value	Heterogeneity (*P*, *I*^*2*^ (%))
Overall survival	21	1.88	1.42–2.50	< 0.01	0.013, 45.4
Country					
China	6	1.81	1.35–2.44	< 0.01	0.172, 35.3
Japan	13	2.37	1.59–3.53	< 0.01	0.222, 21.9
Korea	1	3.33	0.38–29.33	0.278	–, –
Turkey	1	0.44	0.19–0.97	0.043	–, –
Cut-off value					
0.25	1	3.33	0.38–29.33	0.278	–, –
0.5	11	1.79	1.03–3.11	0.039	0.022, 52.0
0.75	3	2.52	1.65–3.83	< 0.01	0.335,8.5
0.8	1	6.20	0.80–47.83	0.080	–, –
0.85	1	1.44	0.12–17.49	0.775	–, –
Continuous variable	4	1.69	1.06–2.72	0.027	0.056,60.3
Source of HR					
Reported	14	1.98	1.46–2.68	< 0.01	0.114, 32.6
Estimated	7	1.70	0.86–3.37	0.126	0.010, 64.6
Histology type					
Adenocarcinoma	13	1.68	1.10–2.56	0.016	0.004,59.0
NSCLC	8	1.88	1.41–2.51	< 0.01	0.400,3.9

*HR* hazard ratio, *CI* confidence interval, *NSCLC* non-small cell lung cancer

**Table 3:** Subgroup analysis for DFS/RFS/PFS

	Number of studies	HR	95% CI	*P* value	Heterogeneity (*P*, *I*^*2*^ (%))
Progress-free survival	22	1.42	1.27–1.59	< 0.01	< 0.01, 81.9
Country					
China	10	1.83	1.39–2.42	< 0.01	< 0.01, 87.7
Japan	9	2.92	1.47–5.81	0.002	< 0.01, 72.6
Korea	2	2.35	1.37–4.05	0.278	0.692, 0.0
Turkey	1	0.63	0.23–1.72	0.043	–, –
Cut-off value					
0.25	1	2.04	0.84–4.96	0.116	–, –
0.50	13	2.73	1.63–4.58	< 0.01	< 0.01, 78.7
0.75	2	2.03	1.13–3.66	0.018	0.117,59.3
0.85	1	2.60	0.16–42.12	0.501	–, –
Continuous variable	5	1.46	1.08–1.99	0.015	< 0.01,90.7
Source of HR					
Reported	16	1.36	1.22–1.52	< 0.01	< 0.01, 84.8
Estimated	6	1.85	1.21–2.83	0.004	0.264, 22.6
Histology type					
Adenocarcinoma	15	2.06	1.48–2.87	< 0.01	< 0.01,83.6
NSCLC	7	1.44	1.02–2.02	0.037	< 0.01,78.9

*HR* hazard ratio, *CI* confidence interval, *NSCLC* non-small cell lung cancer

### Sensitivity analysis

**Fig. 3:**
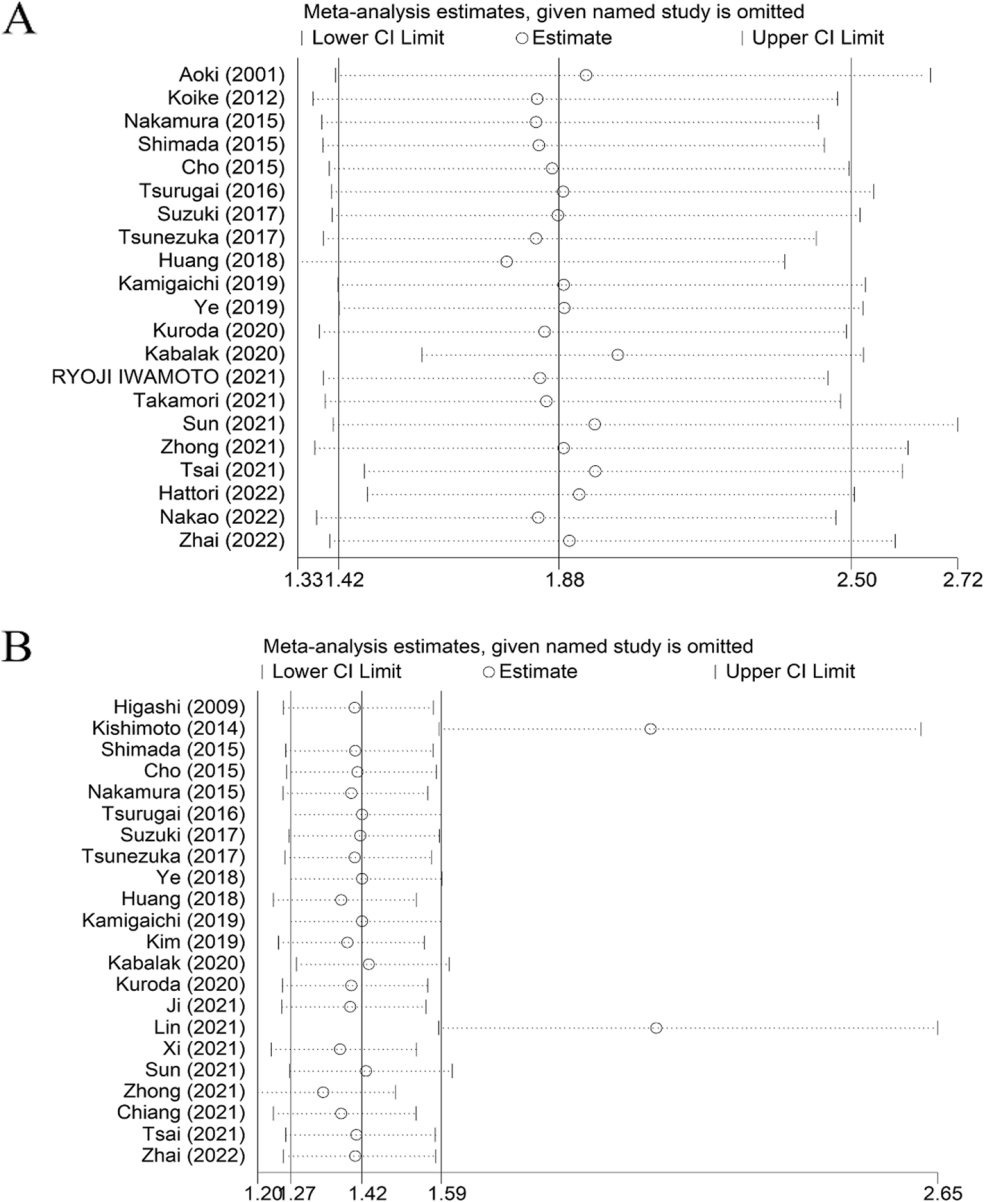
**A** Sensitivity analysis of the relationship between CTR and overall survival. **B** Sensitivity analysis of the relationship between CTR and DFS/RFS/PFS

Sensitivity analysis revealed that the study for OS was stable and reliable (Fig. 3A). However, sensitivity analysis for the study on the relationship between CTR and DFS/RFS/PFS suggested that the studies of Kishimoto et al. [20] Lin et al. [35] and Zhong et al. [39] had a certain impact on our results (Fig. 3B). There was no significant change in the pooled HR (HR = 2.23, 95% CI = 1.69–2.94, *p* < 0.01) or heterogeneity (*I*^2^ = 57.2%, *p* < 0.01) after we discarded these three studies.

### Publication bias

**Fig. 4:**
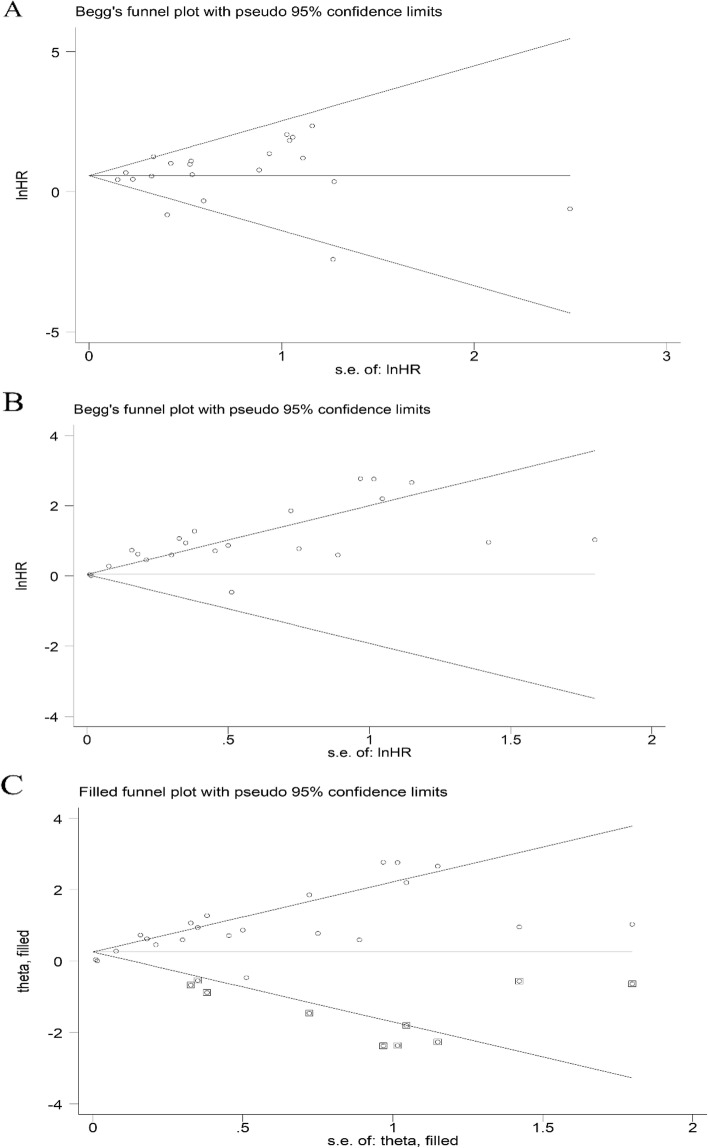
**A** Begg’s funnel plot for the relationship between CTR and overall survival. **B** Begg’s funnel plot for the relationship between CTR and DFS/RFS/PFS. **C** Begg’s funnel plot for the relationship between CTR and DFS/RFS/PFS after using the trim-and-fill method

A symmetrical funnel plot revealed no significant publication bias (*P* > 0.1) in the study for the correlation between CTR and OS (Fig. 4A).

The asymmetrical funnel plot implied significant publication bias for the study on DFS/RFS/PFS. Ten potentially unpublished studies were found using the trim-and-fill method. After adding the 10 potentially unpublished studies, the pooled HR was 1.29 (95% CI 1.15–1.45, *p* < 0.01) using the random effects model, which indicated that the 10 unpublished studies had no significant effect on the result, indicating that the result was reliable and stable (Fig. 4B, C).

## Discussion

In this study, 29 studies with 10,347 patients were included to analyze the prognostic value of CTR. The results suggested that higher CTR was associated with worse prognosis in NSCLC patients. Subgroup analysis by cut-off value demonstrated that this result was valid when the cut-off value was 0.5 or 0.75 or when CTR was a continuous variable. Simultaneously, subgroup analysis stratified by country implied that CTR could be a prognostic factor for patients with NSCLC from Japan and China. Significant heterogeneity was observed among studies focusing on DFS/RFS/PFS as the outcome, warranting cautious interpretation of the findings. The findings from subgroup analysis indicated that the pathology type and source of HR did not exert a significant impact on the statistical significance of the study results. However, within the Korean population and when employing the CTR cut-off values of 0.25 and 0.85, the results were not significant, which may be related to the small number of studies. Given the observed heterogeneity, future studies are recommended to investigate the prognostic significance of CTR in different national populations and the optimal cut-off value of CTR.

An increasing number of studies have found that some CT-based radiomics signatures can be used to predict tumor aggressiveness and prognosis [43, 44]. Our study found that CTR can be used to predict the prognosis of NSCLC patients, and this factor can be included in future research on the prognostic model construction of NSCLC. Nguyen et al. found that the use of imaging features combined with clinical information can improve the accuracy of predicting epidermal growth factor receptor (EGFR) mutation status in patient with NSCLC [45]. Due to our study's limitation in lacking information on EGFR mutations within different CTR groups, we failed to elucidate the relationship between CTR and EGFR mutations, future investigations should be undertaken to address this issue. Lin et al. [35] demonstrated that a higher CTR subgroup had more invasive adenocarcinomas, lymphovascular invasion, and visceral pleural invasion than the lower CTR subgroup. Ono et al. [46] conducted a study to evaluate the association between CTR and immune-related factors, and found that small-sized lung adenocarcinoma with high CTR might be correlated with immunosuppressive conditions in the antitumor immune response compared with low CTR. The results of the above studies may be the reason why NSCLC patients with high CTR have a worse prognosis than those with low CTR, but more high-quality research is needed. Based on the above findings, we believe that CTR can be used to guide the preoperative decision-making of patients with NSCLC, and more aggressive surgical methods and more aggressive adjuvant therapy after surgery may be required for patients with higher CTR.

Although the prognostic value of CTR in NSCLC has been proven by many studies, consensus on the cut-off value of CTR has not yet been reached. Based on our results, when the cut-off value was 0.5 or 0.75 or when CTR was a continuous variable, CTR could predict prognosis. Huang et al. [27] showed that a GGO ratio greater than 75%, conversely, means that a CTR less than 25%, has value in predicting a favorable prognosis in resected lung adenocarcinoma patients. RYOJI IWAMOTO et al. [33] performed an ROC analysis to find the appropriate cut-off value of the CT solid score, which was equal to the CTR. They found that when the cut-off value was 80%, the area under the curve (AUC) for predicting recurrence had the highest sensitivity and specificity. Multivariate analysis indicated that a CT solid score > 80% was associated with an elevated likelihood of recurrence. However, in our studies, no obvious survival differences were observed between the low CTR and high CTR groups when the cut-off value was 0.8 or 0.85. Most of the studies we included used 0.5/0.75 as a cut-off, which limited our further analysis of the prognostic value of CTR with different cut-off values. Therefore, further studies are needed to confirm the appropriate cut-off to predict the prognosis of NSCLC patients.

According to previous studies, the survival and clinicopathological characteristics of part-solid nodules (PSNs) differ from those of pure solids [47]. Therefore, some experts suggested that NSCLC manifesting as PSNs should be treated as a special subtype. Most of our included studies included pure GGOs or pure solids, which each represent a different prognosis than PSNs. We could not perform subgroup analysis to analyze the prognosis of CTR in PSNs due to the lack of studies on PSNs. Kim et al. [13] demonstrated that CTR was not an independent prognostic factor for part-solid lung adenocarcinomas from cT1mi to cT1c. Fu et al. [48] found that a higher CTR indicated worse survival in NSCLC patients with part-solid nodules excluding lepidic pattern–predominant adenocarcinoma. The different conclusions of these two studies remind us that the predictive value of CTR in PSNs is worthy of further study.

It was reported by Pan et al.’s meta-analysis that no significant difference in DFS was found between patients with higher and lower GGO ratios in pathological stage I pulmonary adenocarcinoma [49], which was not consistent with our results. However, only four studies were included in their study, and some studies used the tumor shadow disappearance rate (TDR) [50], which was calculated as the ratio between the tumor area in the mediastinal window setting and that in the lung window setting, to calculate the GGO ratio. Our study not only unified the definition of CTR, but also included more references, and conducted subgroup analysis on different cut-off values. Therefore, our results may be more convincing.

There were still several limitations in our study. First, all included studies were retrospective observational studies, which might cause potential selection and publication bias. Second, the potential impact of our results may be influenced by the unequal distribution of disease stages and treatments among groups, warranting further clarification. Third, HR information for some studies was extracted from Kaplan‒Meier curves, which may generate bias. Fourth, all of the patients included in this study were from China, Japan, Korea, and Turkey, which may limit the generalizability of our findings to other populations and ethnics. Fifth, as a result of a lack of detailed baseline data, such as age, TNM stage, and sex, we could not perform subgroup analyses by these factors. Sixth, significant heterogeneity was observed in our study, and we failed to find the source of the heterogeneity. Seventh, the lack of molecular marker information in our study may have affected our ability to comprehensively analyze the predictive value of CTR.

According to our study, CTR is a good prognostic factor for NSCLC patients, but further studies need to be conducted to verify this.

## Supplementary Information

**Additional file 1. **

**Additional file 2.** **Supplementary Table S1.** Search strategy. **Supplementary Table S2.** NOS score. **Figure S1.** Measurement of CTR, CTR was defined as the maximum size of consolidation to the maximum tumor size in the lung window on computed tomography of the chest with or without IV contrast. CTR, consolidation to tumor ratio. **Figure S2.** Forest plot for the relationship between CTR and overall survival in stage I patients. CTR, consolidation to tumor ratio. **Figure S3. **Forest plot for the relationship between CTR and DFS/RFS/PFS in stage I patients. CTR, consolidation to tumor ratio; DFS, disease-free survival; RFS, recurrence-free survival; PFS, progression-free survival.

## Data Availability

Not applicable.

## Abbreviations

CT: Computed tomography
CTR: Consolidation to tumor ratio
CI: Confidence interval
DFS: Disease-free survival
EGFR: Epidermal growth factor receptor
HR: Hazard ratio
NSCLC: Non-small cell lung cancer
NOS: Newcastle‒Ottawa quality assessment scale
OS: Overall survival
PFS: Progression-free survival
PROSPERO: The International Prospective Registry of Systematic Reviews
PSN: Part-solid nodule
RFS: Recurrence-free survival
SUV max: Maximal standardized uptake value
TDR: Tumor shadow disappearance rate
