# Supplementary material for: Prognostic value of consolidation-to-tumor ratio on computed tomography in NSCLC: a meta-analysis
Source: World J Surg Oncol. 2023 Jun 22;21:190. doi: 10.1186/s12957-023-03081-y (PMC10286506; doi:10.1186/s12957-023-03081-y)
Supplement: Supplementary file 1 — Additional file 1. [file 12957_2023_3081_MOESM1_ESM.docx]

| Authors | Year | Study period | Country | Sample size | MFP | Study type | Age(years) | Male | Female | Histology type | Cutoff | Endpoint | Source of HR | NOS | Stage | Treatment |
| --- | --- | --- | --- | --- | --- | --- | --- | --- | --- | --- | --- | --- | --- | --- | --- | --- |
| Aoki et al | 2001 | 1990-1999 | Japan | 127 | 37.1 | Retro | Mean64,range:32-84 | 68 | 59 | ADA | 0.5 | OS | E | 7 | I | Surgery |
| Higashi et al | 2009 | 1997-2005 | Japan | 87 | 18 | Retro | Mean64,range:42-84 | 40 | 47 | ADA | 0.5 | DFS | E | 7 | I-III | Surgery |
| Koike et al | 2012 | 1992-2009 | Japan | 223 | 70 | Retro | Median65,range42-81 | 108 | 115 | NSCLC | 0.75 | OS | R | 8 | I-II | Surgery |
| Kishimoto et al | 2014 | 2006-2010 | Japan | 169 | 42 | Retro | Median71,range34-87 | 114 | 55 | NSCLC | C | DFS | R | 8 | I-IV | Surgery |
| Nakamura et al | 2015 | 2005-2011 | Japan | 113 | 46 | Retro | Median71,range56-79:25;Median68,range26-85:88 | 64 | 49 | ADA | 0.5 | OS/DFS | R | 8 | I | Surgery |
| Shimada et al | 2015 | 2004-2010 | Japan | 67 | 58.9 | Retro | Median69 (IQR60–75) | 22 | 45 | NSSLC | 0.5 | OS/RFS | R | 8 | I-III | Surgery |
| Cho et al | 2015 | 2001-2010 | Korea | 97 | 44.7 | Retro | Median62,range36-81 | 43 | 54 | ADA | 0.25 | OS/RFS | E | 8 | I | Surgery |
| Tsurugai et al | 2016 | 2005-2014 | Japan | 155 | 34.7 | Retro | Median77 (IQR72–81) | 99 | 56 | NSCLC | 0.5 | OS/DFS | R | 8 | I | SBRT |
| Suzuki et al | 2017 | 2003-2007 | Japan | 392 | 84 | Retro | <70:283; ≥70:109 | 181 | 211 | ADA | 0.5 | OS/RFS | E | 7 | I | Surgery |
| Tsunezuka et al | 2017 | 2008-2012 | Japan | 62 | 55.1 | Retro | Mean74,range:55-91 | 42 | 20 | NSCLC | 0.5 | OS/RFS | R | 7 | I-IV | Surgery |
| Huang et al | 2018 | 2004-2013 | China | 789 | 87 | Retro | Mean ± SD, 59.94 ± 9.45:267; 62.57 ± 10.99；522 | 258 | 531 | ADA | 0.75 | OS/DFS | E | 7 | 0-IV | Surgery |
| Ye et al | 2018 | 2008-2014 | China | 736 | 38 | Retro | Median56,range26-82 | NA | NA | ADA | C | RFS | R | 7 | NA | Surgery |
| Kamigaichi et al | 2019 | 2007-2016 | Japan | 166 | 49.3 | Retro | Median67 (IQR,61-72) | 86 | 80 | NSCLC | 0.85 | OS/RFS | E | 7 | 0-I | Surgery |
| Kim et al | 2019 | 2009-2015 | Korea | 691 | 39 | Retro | Median63 (IQR,55-70) | 281 | 410 | ADA | 0.5 | DFS | R | 7 | I | Surgery |
| Ye et al | 2019 | 2008-2014 | China | 329 | 42.2 | Retro | Mean ± SD, 58.89 ± 9.71 | 91 | 224 | ADA | 0.5 | OS | R | 8 | I | Surgery |
| Kuroda et al | 2020 | 2006-2010 | Japan | 260 | 83.8 | Retro | Mean64,range:32-84 | 128 | 132 | NSCLC | 0.5 | OS/DFS | R | 7 | I | Surgery |
| Kabalak et al | 2020 | 2013-2016 | Turkey | 156 | 40 | Retro | Mean ± SD, 62.08 ± 8.7 | 121 | 35 | ADA | 0.5 | OS/PFS | E | 8 | I | Surgery |
| RYOJI IWAMOTO et al.(34) | 2021 | 2000-2009 | Japan | 73 | 77 | Retro | Median66,range45-85 | 39 | 34 | ADA | 0.8 | OS | R | 7 | NA | Surgery |
| Takamori et al | 2021 | 2006-2014 | Japan | 85 | 87.6 | Retro | Median64 (IQR,58.5–72) | 36 | 49 | NSCLC | 0.5 | OS | E | 7 | 0-I | Surgery |
| Sun et al | 2021 | 2014.01-2014.12 | China | 257 | 76 | Retro | Mean59 | 109 | 148 | NSCLC | C | OS/RFS | R | 7 | I-III | Surgery |
| Zhong et al | 2021 | 2011-2012 | China | 620 | 72.4 | Retro | Mean ± SD,59.6± 8.2 | 269 | 351 | ADA | C | OS/RFS | R | 7 | I | Surgery |
| Ji et al | 2021 | 2014-2015 | China | 190 | 51 | Retro | Mean61,range:52-69 | 66 | 124 | ADA | 0.5 | PFS | R | 7 | I-III | Surgery |
| Lin et al | 2021 | 2013-2015 | China | 372 | 55 | Retro | Mean ± SD, 59.7±10.5 | 113 | 259 | ADA | 0.5 | RFS | R | 7 | I | Surgery |
| Xi et al | 2021 | 2011-2016 | China | 862 | 47 | Retro | Median59 | 254 | 608 | ADA | C | RFS | R | 7 | I | Surgery |
| Chiang et al | 2021 | 2011-2017 | China | 1002 | 43.2 | Retro | Mean ± SD,59.6± 11.8 | 323 | 679 | ADA | 0.5 | DFS | R | 8 | 0-III | Surgery |
| Tsai et al | 2021 | 2003-2015 | China | 149 | 74 | Retro | Mean ± SD,62± 10.8 | 54 | 95 | ADA | 0.5 | OS/DFS | R | 8 | NA | Surgery |
| Hattori et al | 2022 | 2008-2017 | Japan | 603 | 54 | Retro | Mean ± SD,69.1± 10.8:120; 68.2± 10.3:483 | 348 | 255 | ADA | C | OS | R | 8 | I-III | Surgery |
| Nakao et al | 2022 | 2010-2017 | Japan | 1014 | 61 | Retro | Median67 (IQR,61-73):717; median66 (IQR,59–72):297 | 445 | 569 | ADA | C | OS | R | 8 | I-III | Surgery |
| Zhai et al | 2022 | 2008-2018 | China | 501 | 64.8 | Retro | Median61,range30-82 | 253 | 248 | ADA | 0.75 | OS/DFS | R | 8 | I-II | Surgery |
| Basic Characteristics of Included Studies. MFP, median follow-up time; HR, hazard ratio; NOS, Newcastle–Ottawa scale; NSCLC, non-small cell lung cancer; Retro, retrospective; ADA, adenocarcinoma;  C, continuous; OS, overall survival; DFS, disease-free survival; RFS, recurrence-free survival; PFS, progression-free survival; E, estimated; R, reported; NA, not available; SBRT, stereotactic body radiation therapy; IQR, interquartile range; SD, standard deviation. | | | | | | | | | | | | | | | | |
