# Supplementary material for: Prognostic value of consolidation-to-tumor ratio on computed tomography in NSCLC: a meta-analysis
Source: World J Surg Oncol. 2023 Jun 22;21:190. doi: 10.1186/s12957-023-03081-y (PMC10286506; doi:10.1186/s12957-023-03081-y)
Supplement: Supplementary file 2 — Additional file 2. Supplementary Table S1. Search strategy. Supplementary Table S2. NOS score. Figure S1. Measurement of CTR, CTR was defined as the maximum size of consolidation to the maximum tumor size in the lung window on computed tomography of the chest with or without IV contrast. CTR, consolidation to tumor ratio. Figure S2. Forest plot for the relationship between CTR and overall survival in stage I patients. CTR, consolidation to tumor ratio. Figure S3. Forest plot for the relationship between CTR and DFS/RFS/PFS in stage I patients. CTR, consolidation to tumor ratio; DFS, disease-free survival; RFS, recurrence-free survival; PFS, progression-free survival. [file 12957_2023_3081_MOESM2_ESM.docx]

**Supplementary Table S1. Search strategy.**

| **Pubmed(n=1428)** |
| --- |
| ((((((cancer) OR (carcinoma)) OR (tumor)) OR (neoplasm)) AND ((lung) OR (pulmonary))) OR ((((part-solid) OR (sub-solid)) OR (ground-glass opacity)) OR (ground-glass nodule))) AND (((((((((consolidation-to-tumour ratio) OR (C/T ratio)) OR (consolidation to tumour ratio)) OR (solid component ratio)) OR (CT solid score)) OR (consolidation tumor ratio)) OR (consolidation/tumor ratio)) OR (percentage of solid component)) OR (solid component)) |
| **Embase(n=648)** |
| #1 (lung or pulmonary).af.  #2 (tumor or cancer or carcinoma or neoplasm) .af.  #3 (part-solid or sub-solid or ground-glass opacity or ground-glass nodule) .af.  #4 #1 AND #2  #5 #3 OR #4  #6 (consolidation to tumor ratio or solid component or consolidation tumor ratio or CT solid score) .af.  #7 #5 AND #6 |
| **Web of Science(n=2799)** |
| # 1 TOPIC: (canccer)  # 2 TOPIC: (carcinoma)  # 3 TOPIC: (tumor)  # 4 TOPIC: (neoplasm)  # 5 #1 OR #2 OR #3 OR #4  # 6 TOPIC: (lung)  # 7 TOPIC: (pulmonary)  # 8 #6 OR #7  # 9 #5 AND #8  # 10 TOPIC: (part-solid)  # 11 TOPIC: (sub-solid)  # 12 TOPIC: (ground-glass opacity)  # 13 TOPIC: (ground-glass nodule)  # 14 #10 OR #11 OR #12 OR #13  #15 #14 OR #9  #16 ((((TS=(consolidation to tumour ratio)) OR TS=(consolidationtumor ratio)) OR TS=(consolidation/tumor ratio)) OR TS=(C/Tratio)) OR TS=(solid component)  # 17 #15 AND #16 |

**Supplementary Table S2.NOS score.**

| **Study** | **Selection** | | | | **Comparability of cases and controls on the basis of the design or analysis ^a^** | **Exposure** | | | **Scores** | |
| --- | --- | --- | --- | --- | --- | --- | --- | --- | --- | --- |
|  | **Representativeness of the exposed cohort** | **Selection of the non-exposed cohort** | **Ascertainment of exposure** | **Outcome not present at the start of study** |  | **Ascertainment of outcome** | **Follow-up long enough for outcomes to occur** | **Adequacy of follow-up** | |  |
| Aoki 2001 | ★ | ★ | ★ |  | ★ | ★ | ★ | ★ | | 7 |
| Higashi 2009 | ★ | ★ | ★ |  | ★ | ★ | ★ | ★ | | 7 |
| Koike 2012 | ★ | ★ | ★ |  | ★★ | ★ | ★ | ★ | | 8 |
| Kishimoto 2014 | ★ | ★ | ★ |  | ★★ | ★ | ★ | ★ | | 8 |
| Nakamura 2015 | ★ | ★ | ★ |  | ★★ | ★ | ★ | ★ | | 8 |
| Shimada 2015 | ★ | ★ | ★ |  | ★★ | ★ | ★ | ★ | | 8 |
| Cho 2015 | ★ | ★ | ★ |  | ★★ | ★ | ★ | ★ | | 8 |
| Tsurugai 2016 | ★ | ★ | ★ |  | ★★ | ★ | ★ | ★ | | 8 |
| Suzuki 2017 | ★ | ★ | ★ |  | ★ | ★ | ★ | ★ | | 7 |
| Tsunezuka 2017 | ★ | ★ | ★ |  | ★ | ★ | ★ | ★ | | 7 |
| Huang 2018 | ★ | ★ | ★ |  | ★ | ★ | ★ | ★ | | 7 |
| Ye 2018 | ★ | ★ | ★ |  | ★ | ★ | ★ | ★ | | 7 |
| Kamigaichi 2019 | ★ | ★ | ★ |  | ★ | ★ | ★ | ★ | | 7 |
| Kim 2019 | ★ | ★ | ★ |  | ★ | ★ | ★ | ★ | | 7 |
| Ye 2019 | ★ | ★ | ★ |  | ★★ | ★ | ★ | ★ | | 8 |
| Kuroda 2020 | ★ | ★ | ★ |  | ★ | ★ | ★ | ★ | | 7 |
| Kabalak 2020 | ★ | ★ | ★ |  | ★★ | ★ | ★ | ★ | | 8 |
| RYOJI IWAMOTO 2021 | ★ | ★ | ★ |  | ★★ | ★ | ★ | ★ | | 7 |
| Takamori 2021 | ★ | ★ | ★ |  | ★ | ★ | ★ | ★ | | 7 |
| Sun 2021 | ★ | ★ | ★ |  | ★ | ★ | ★ | ★ | | 7 |
| Zhong 2021 | ★ | ★ | ★ |  | ★ | ★ | ★ | ★ | | 7 |
| Ji 2021 | ★ | ★ | ★ |  | ★ | ★ | ★ | ★ | | 7 |
| Lin 2021 | ★ | ★ | ★ |  | ★ | ★ | ★ | ★ | | 7 |
| Xi 2021 | ★ | ★ | ★ |  | ★ | ★ | ★ | ★ | | 7 |
| Chiang 2021 | ★ | ★ | ★ |  | ★★ | ★ | ★ | ★ | | 8 |
| Tsai 2021 | ★ | ★ | ★ |  | ★★ | ★ | ★ | ★ | | 8 |
| Hattori 2022 | ★ | ★ | ★ |  | ★★ | ★ | ★ | ★ | | 8 |
| Nakao 2022  Zhai 2022 | ★  ★ | ★  ★ | ★  ★ |  | ★★  ★★ | ★  ★ | ★  ★ | ★  ★ | | 8  8 |

^a^A maximum of 2 stars can be allotted in this category, one for age, the other for other controlled factors.


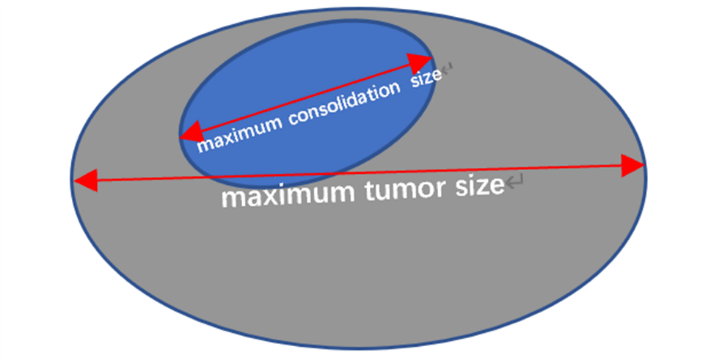


**Figure S1.** Measurement of CTR, CTR was defined as the maximum size of consolidation to the maximum tumor size in the lung window on computed tomography of the chest with or without IV contrast. CTR, consolidation to tumor ratio.


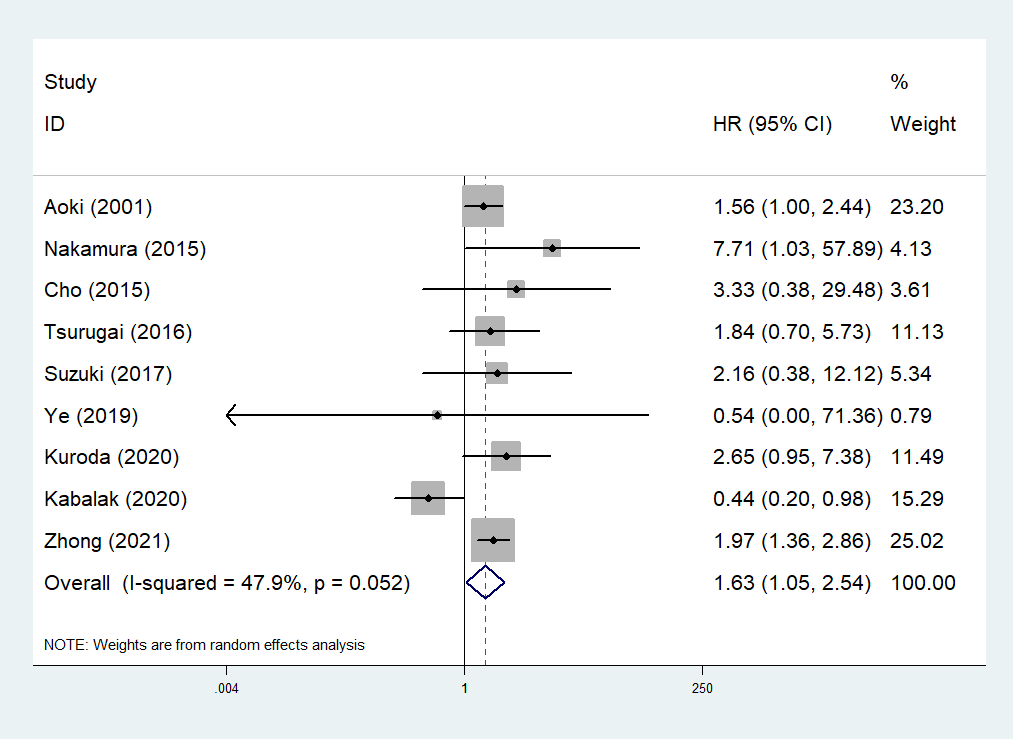


**Figure S2**. Forest plot for the relationship between CTR and overall survival in stage I patients. CTR, consolidation to tumor ratio.


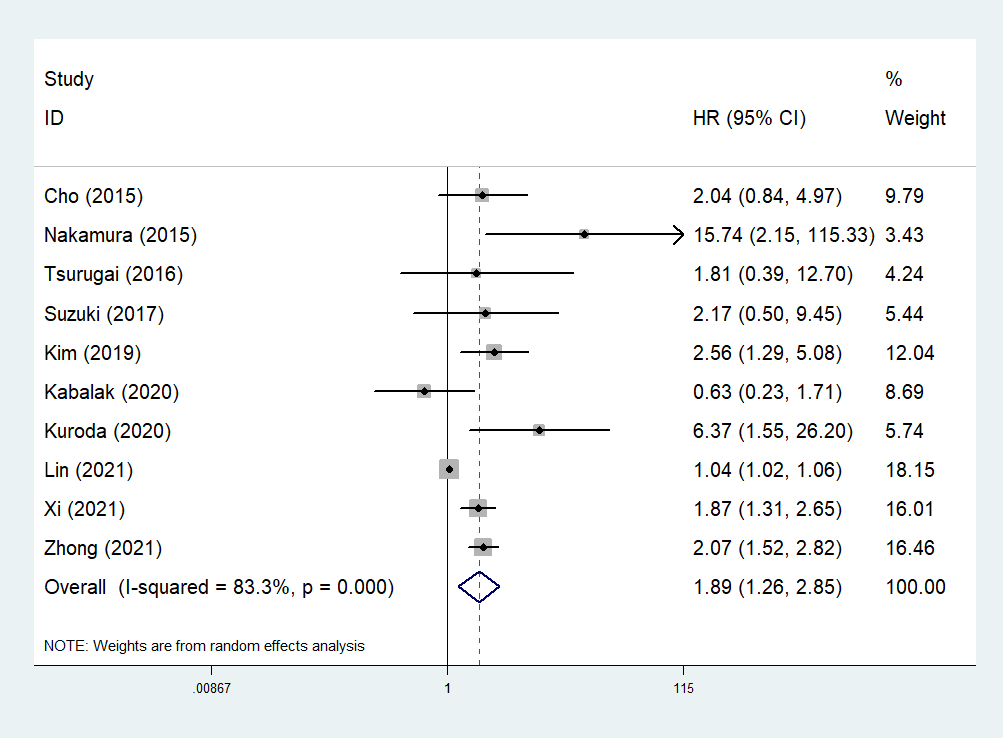


**Figure S3.** Forest plot for the relationship between CTR and DFS/RFS/PFS in stage I patients. CTR, consolidation to tumor ratio; DFS, disease-free survival; RFS, recurrence-free survival; PFS, progression-free survival.
